# A Sjögren’s syndrome patient rapidly progressed to scleroderma renal crisis after COVID-19 infection

**DOI:** 10.1093/rap/rkad107

**Published:** 2023-12-09

**Authors:** Shenyi Yu, Fangling Yao, Fang Li, Zhaohui Deng, Li Deng

**Affiliations:** Department of Rheumatology and Immunology, Zhuzhou Hospital Affiliated to Xiangya Medical College, Central South University, Zhuzhou, Hunan, China; Department of Rheumatology and Immunology, Zhuzhou Hospital Affiliated to Xiangya Medical College, Central South University, Zhuzhou, Hunan, China; Department of Radiology, Zhuzhou Hospital Affiliated to Xiangya Medical College, Central South University, Zhuzhou, Hunan, China; Department of Rheumatology and Immunology, Zhuzhou Hospital Affiliated to Xiangya Medical College, Central South University, Zhuzhou, Hunan, China; Department of Rheumatology and Immunology, Zhuzhou Hospital Affiliated to Xiangya Medical College, Central South University, Zhuzhou, Hunan, China

Key message

- The original connective tissue disease may change after infection with COVID-19.

Dear Editor, A 67-year-old woman presented with dryness of the mouth and eyes for 14 years. She had no relevant family history. In 2016, the patient visited our hospital and tested positive for ANA and anti-SSA antibody. Anti-Scl-70 antibody was negative. At that time, the patient had no RP or skin sclerosis manifestations. Her labial salivary gland biopsy revealed the infiltration of plasma cells and lymphocytes around the duct of the minor salivary glands. The patient underwent the unstimulated salivary flow rate test and the result was <0.05 ml/min. She tested positive in the Schirmer’s test and fluorescent dye test. She was diagnosed with SS based on the ACR/EULAR classification criteria. A chest CT scan suggested interstitial lung disease (ILD). The patient received prednisone and mycophenolate mofetil treatment. Her dosage of prednisone gradually decreased to 5 mg/day in April 2017. The patient was not vaccinated with COVID-19 vaccine.

In December 2022, she was infected with COVID-19 for the first time. Haemoglobin was 108 g/l, serum creatinine was 59 μmol/l and CRP was 26.9 mg/l. After receiving symptomatic treatment such as oxygen inhalation and cough relieving, her condition improved and she was discharged. During hospitalization, her blood pressure (BP) was normal. On 2 March 2023 she developed mild oedema in both lower limbs, mild bilateral eyelid oedema and skin sclerosis on the face and hands, accompanied by significant RP. On 17 March 2023, her BP at admission was 154/88 mmHg. She had an elevated BP of 185/112 mmHg in 48 h. She was treated with a sufficient amount of captopril immediately. Haemoglobin was 73 g/l, platelet count was 101 × 10^9^/l and blood albumin was 35.8 g/l. Serum creatinine was 303 μmol/l and urea nitrogen was 17.59 mmol/l. Urinalysis indicated proteinuria (1+) and occult blood (2+). CRP was 35.8 mg/l and ESR was 90 mm/h. Lactate dehydrogenase was 469 U/l and creatinine kinase was normal. ANA was positive at a titre of 1:320. Anti-Scl-70 antibody and anti-Ku antibody were strongly positive. Anti-RNA polymerase III antibodies and anti-centromere antibody were negative. ANCA, anti-GBM antibodies and aPL antibodies were negative. Complement was normal. The glycated haemoglobin was 4.9%. Peripheral blood smear showed schistocytes and a direct Coombs test was negative. Chest high-resolution CT scan suggested ILD, oesophageal dilation, bilateral pleural effusion and a small amount of pericardial effusion (Fig. 1). Renal ultrasound showed regular morphology and normal size of both kidneys, with diffuse parenchymal lesions in both kidneys and clear boundaries between the cortex and medulla. Renal vascular ultrasound did not show renal vascular thrombosis. We suggested that she complete a kidney biopsy, but after consideration, the patient and her family refused. She was diagnosed with diffuse SSc with scleroderma renal crisis (SRC). Her renal function rapidly deteriorated, with serum creatinine reaching 614 μmol/l and her urine output gradually decreased until anuria. On 22 March 2023, the patient began receiving haemodialysis treatment. After receiving the above-mentioned treatment, her BP returned to normal and regular haemodialysis is still necessary. The patient is still undergoing regular follow-up.

**Figure 1.**
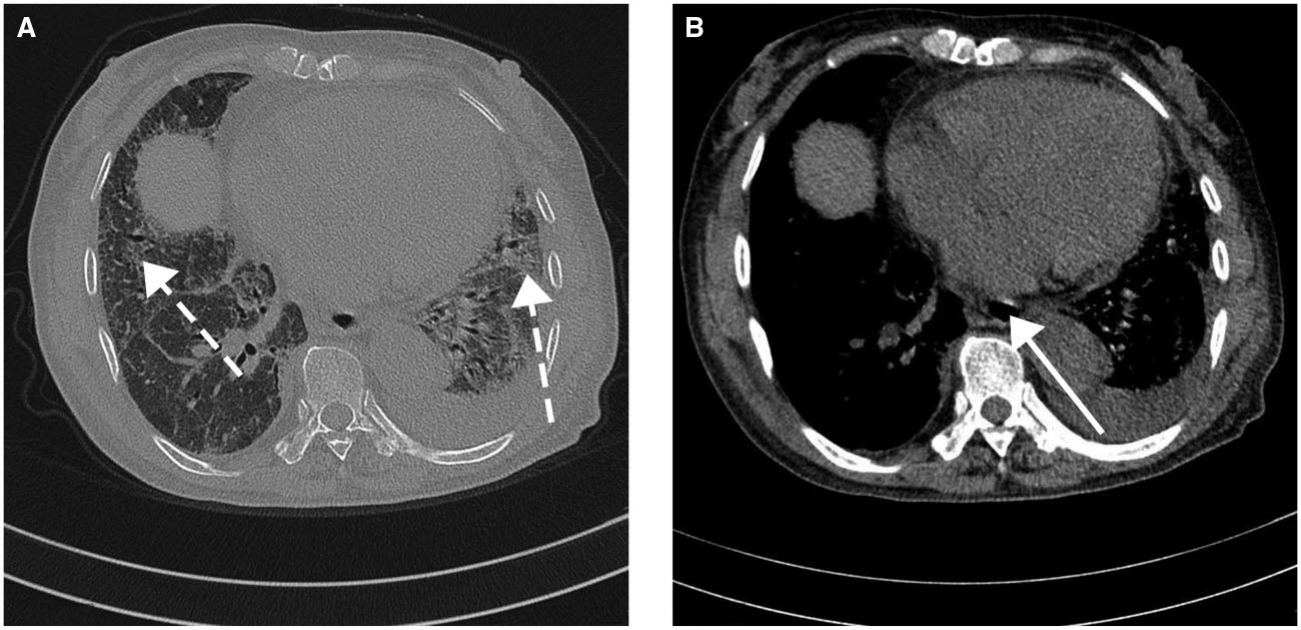
High-resolution CT (HRCT) of the chest. Chest HRCT images showed subpleural ground-glass shadows and grid-like lesions compatible with ILD. Oesophageal dilation, traction bronchiectasis, bilateral pleural effusion and a small amount of pericardial effusion were present. **(A)** Chest HRCT, pulmonary window. White broken arrows indicate ILD. **(B)** Chest HRCT, mediastinal window. White arrow indicates oesophageal dilation

SSc is a complex multisystem CTD that can lead to skin and visceral fibrosis [1]. The most fatal complication of SSc is SRC, with an incidence rate of 2–3% [2]. At present, there are no reports of overlapping SSc on the basis of SS and rapidly developing into SRC after infection with COVID-19. The patient we reported was previously diagnosed as SS. After COVID-19 infection, the patient experienced rapid progress of skin thickening in a short period of time, new anaemia, RP, significantly increased BP, rapidly deteriorated renal function and changed antibody spectrum. Based on the patient’s clinical manifestations, laboratory tests and imaging examinations, we consider that the patient had SSc on the basis of SS. Several common aspects of the pathophysiology of SSc and COVID-19 infection, including the release of cytokines such as IL-6, a hypercoagulable state, endothelial activation and vasculopathy, may explain its possible role as a trigger for SRC [3]. Jalalzadeh *et al.* [4] reported a patient with SSc who suffered from new renal failure after COVID-19 infection. Her kidney biopsy showed that she had ANCA-associated vasculitis, while perinuclear ANCA and myeloperoxidase antibodies were positive [4]. But our patient’s ANCA was negative, even though she refused to undergo a kidney biopsy. After infection with COVID-19, patients may have new autoimmune diseases without underlying diseases [5]. If the patient has previous CTD, the original CTD may also change after infection with COVID-19 [4]. Patients with CTD infected with COVID-19 should pay attention to detection of the autoantibody spectrum and the assessment of organ involvement in case of new clinical symptoms that are difficult to explain in basic diseases, so as to avoid a missed diagnosis and delayed treatment.

## Data Availability

The data underlying this article are available in the article.
